# Complete Genome Assemblies of All *Xanthomonas translucens* Pathotype Strains Reveal Three Genetically Distinct Clades

**DOI:** 10.3389/fmicb.2021.817815

**Published:** 2022-03-02

**Authors:** Florian Goettelmann, Veronica Roman-Reyna, Sébastien Cunnac, Jonathan M. Jacobs, Claude Bragard, Bruno Studer, Ralf Koebnik, Roland Kölliker

**Affiliations:** ^1^Molecular Plant Breeding, Institute of Agricultural Sciences, ETH Zürich, Zurich, Switzerland; ^2^Department of Plant Pathology, The Ohio State University, Columbus, OH, United States; ^3^Infectious Diseases Institute, The Ohio State University, Columbus, OH, United States; ^4^Plant Health Institute of Montpellier, University of Montpellier, CIRAD, INRAE, Institut Agro, IRD, Montpellier, France; ^5^Earth and Life Institute, UCLouvain, Louvain-la-Neuve, Belgium

**Keywords:** *Xanthomonas translucens*, complete genomes, phylogeny, host adaptation, comparative genomics, virulence factors

## Abstract

The *Xanthomonas translucens* species comprises phytopathogenic bacteria that can cause serious damage to cereals and to forage grasses. So far, the genomic resources for *X. translucens* were limited, which hindered further understanding of the host–pathogen interactions at the molecular level and the development of disease-resistant cultivars. To this end, we complemented the available complete genome sequence of the *X. translucens* pv. *translucens* pathotype strain DSM 18974 by sequencing the genomes of all the other 10 *X. translucens* pathotype strains using PacBio long-read technology and assembled complete genome sequences. Phylogeny based on average nucleotide identity (ANI) revealed three distinct clades within the species, which we propose to classify as clades Xt-I, Xt-II, and Xt-III. In addition to 2,181 core *X. translucens* genes, a total of 190, 588, and 168 genes were found to be exclusive to each clade, respectively. Moreover, 29 non-transcription activator-like effector (TALE) and 21 TALE type III effector classes were found, and clade- or strain-specific effectors were identified. Further investigation of these genes could help to identify genes that are critically involved in pathogenicity and/or host adaptation, setting the grounds for the development of new resistant cultivars.

## Introduction

*Xanthomonas translucens* is a species of Gram-negative phytopathogenic bacteria that causes serious damage in gramineous plants. To date, a total of 11 pathovars have been defined based on their host range, each represented by one strain referred to as the pathotype strain and deposited in the appropriate bacterial strain collections. Two major groups are historically distinguished within the species: the “translucens” group and the “graminis” group. The translucens group consists of pathovars *cerealis*, *hordei*, *secalis*, *translucens*, and *undulosa*, which cause leaf streak and black chaff in economically important cereals such as wheat (*Triticum* spp.) and barley (*Hordeum vulgare*) (Sapkota et al., 2020). The graminis group consists of pathovars *arrhenatheri*, *graminis*, *phlei*, *phleipratensis*, and *poae*, which cause bacterial wilt in forage grasses (Egli et al., 1975; Egli and Schmidt, 1982; Stead, 1989). Although recent genome sequence data loosely supported this phenotypic classification (Peng et al., 2016; Hersemann et al., 2017; Langlois et al., 2017; Shah et al., 2021), these groups are not clearly defined based on the genetic relationships between the strains. Moreover, genomic studies have previously suggested that pv. *cerealis* could be genetically distinct from the other “translucens” group pathovars (Bragard et al., 1997; Rademaker et al., 2006; Peng et al., 2016; Langlois et al., 2017). Furthermore, *Xanthomonas* strains causing dieback in pistachio (*Pistacia vera* L.) were found to belong to the *X. translucens* species, a species so far thought to infect only monocotyledonous plants (Giblot-Ducray et al., 2009). These strains were classified as pv. *pistaciae*, in which two groups were distinguished by rep-PCR and *gyrB*-based phylogeny and are referred to as groups A and B (Facelli et al., 2005; Marefat et al., 2006; Giblot-Ducray et al., 2009). The group A strain was found to be closely related to pathovars *translucens*, *secalis*, and *undulosa*, while the group B strain was closest to pv. *cerealis*, suggesting that the ability to infect pistachio may have evolved two separate times in *X. translucens*.

*X. translucens* pathovars were initially classified solely based on their host range as pathovars of the *X. campestris* species. However, a classification based on DNA–DNA hybridization was proposed by Vauterin et al. (1995), who amended these pathovars of *X. campestris* to the species level, forming the *X. translucens* species. Nonetheless, though these pathovars are now considered pathovars of the *X. translucens* species, the definition of these pathovars is still based on their host range as before. Phylogeny is crucial to better understand the evolutionary history of each pathovar, in order to determine how each strain has adapted to infect its hosts. This knowledge is key in defining targets for breeding resistant cultivars. The current definition of pathovars of *X. translucens* might not properly reflect these processes of host adaptation, and a better definition based on the genetic relationships between the strains would improve the comprehension of the host–pathogen relationships. Recent advances in sequencing technology now allow for the rapid sequencing of complete genomes of bacterial strains, which provide a better basis for phylogenetic analyses. At the time of this study, complete genome sequences were publicly available for pv. *cerealis* strain NXtc01 (Shah et al., 2019); pv. *translucens* strains DSM 18974 (Jaenicke et al., 2016), XtKm7, XtKm8, XtKm9, XtKm33, XtKm34 (Shah et al., 2021); and pv. *undulosa* strains XT4699 (Peng et al., 2016), ICMP 11055 (Falahi Charkhabi et al., 2017), LW16, P3 (Peng et al., 2019), XtFa1, XtLr8, XtKm12, and XtKm15 (Shah et al., 2021). Though the number of available complete genome sequences is continuously growing, these sequences represent only three pathovars to date, with a complete genome sequence being available for only one pathotype strain.

High-quality genome sequences also help in the identification of genes that are directly linked to the pathogen’s virulence and host range. Indeed, successful infection by pathogenic bacteria is mediated by a set of virulence factors, including degradative enzymes and effector proteins. These are often host specific, and the repertoire of virulence factors that a bacterial strain possesses defines which hosts it is able to infect. In *Xanthomonas* species, virulence factors generally depend on the type II and type III secretion systems (T2SS and T3SS, respectively). These secretion systems allow the bacteria to export virulence factors in order to enable or facilitate their proliferation and survival in the host by targeting specific host components (Büttner and Bonas, 2010; Alvarez-Martinez et al., 2021). Identifying which virulence factors are involved in the pathogen’s virulence and which host components are the targets of these virulence factors is crucial, as these host components can then be the focus of resistance breeding.

The T3SS is encoded by the “hypersensitive reaction and pathogenicity” (*hrp*) gene cluster (Bonas et al., 1991). Some *hrp* genes that are highly conserved are referred to as “*hrp*-conserved” (*hrc*) genes (Bogdanove et al., 1996). Moreover, some genes in the cluster that are involved in, but not necessary to, the host–plant interaction are called “*hrp*-associated” (*hpa*) genes. In the *X. translucens* species, the core *hrp* cluster consists of 23 genes, with 8 *hrp* genes, 11 *hrc* genes, and 4 *hpa* genes (Wichmann et al., 2013; Pesce et al., 2017).

This secretion system injects effector proteins into the host cell. In *Xanthomonas*, these effectors are generally called “*Xanthomonas* outer proteins” (Xop), with 53 classes from XopA to XopBA (White et al., 2009). Other effectors are named according to their avirulence characteristics, causing hypersensitive response in the host, such as AvrBs1 to AvrBs3. These effectors are key virulence factors, as, when translocated into the host cell, they are able to target the different pathways of the host, allowing the pathogen, for example, to acquire nutrients or to evade or suppress host defenses. A specific type of effectors secreted by the T3SS are the “transcription activator-like” effectors (TALEs). Their amino acid sequences contain highly conserved repetitive sequences of ∼34 amino acids, with only the 12th and 13th residues being hypervariable and referred to as the “repeat variable di-residue” (RVD). The RVD array of each TALE allows it to bind to specific nucleotide sequences in the host DNA, thus activating the expression of the neighboring genes to the pathogen’s advantage (Streubel et al., 2017; Wang et al., 2017).

Due to their repetitive sequences, TALE genes are difficult to assemble using short-read sequencing technology. Complete genome sequences based on either long-read sequencing, a very high coverage of short-read sequencing, or a mixture of both, are thus necessary to properly identify these effectors. Such high-quality genome sequences allowed the identification of eight and five TALE genes in pv. *translucens* strains DSM 18974 and UPB886, respectively (Jaenicke et al., 2016; Roman-Reyna et al., 2020), eight and seven in pv. *undulosa* strains XT4699 and ICMP 11055, respectively (Peng et al., 2016; Falahi Charkhabi et al., 2017), and two in pv. *cerealis* strains CFBP 2541 and NXtc01 (Pesce et al., 2015; Shah et al., 2019). However, of these, only four were functionally characterized and found to play a role in virulence to date (Falahi Charkhabi et al., 2017; Peng et al., 2019; Shah et al., 2019). No TALE has yet been identified in other pathovars of *X. translucens*.

Similar to the T3SS, the T2SS is responsible for the export of virulence factors, most of which are cell wall-degradative enzymes, into the host apoplasm (Jha et al., 2005). In *Xanthomonas*, two types of clusters encoding the T2SS can be found. The *xps* cluster is conserved across *Xanthomonas* species, while the *xcs* cluster is only found in some species such as *X. citri* and *X. campestris* (Szczesny et al., 2010).

In addition to the T2SS and T3SS, the type IV and type VI secretion systems (T4SS and T6SS, respectively) also secrete proteins that may affect the virulence of the pathogen. However, while the T2SS and the T3SS target host components, the T4SS and the T6SS are involved in the defense against microbial predators such as amoeba, as well as in the competition with other microorganisms (Büttner and Bonas, 2010; Alvarez-Martinez et al., 2021). The T4SS is evolutionarily related to bacterial conjugation systems and is involved in the competition with other bacteria by injecting protein effectors or protein–DNA complexes into their cells (Sgro et al., 2019). The T6SS is related to the tail of bacteriophages and, similarly to the T4SS, is able to inject effector proteins into prokaryotic as well as eukaryotic cells (Bayer-Santos et al., 2019). In the Xanthomonadales order, three subtypes of T6SS have been found: subtypes 1, 3, and 4. Moreover, subtype 3 is further subdivided into subgroups 3^∗^, 3^∗∗^, and 3^∗∗∗^. Although not directly related to virulence, the T4SS and T6SS could be key elements in the proliferation and survival of the bacteria on the host plant (Souza et al., 2015; Choi et al., 2020).

In this study, in order to complement the available complete genome sequence of the pv. *translucens* pathotype strain DSM 18974 (Jaenicke et al., 2016), we sequenced the whole genomes of all the other 10 pathotype strains of the *X. translucens* species, as well as a representative strain of the *X. translucens* pv. *pistaciae* group B, to produce high-quality genome sequences. Using these, we built a phylogeny of these strains to clarify the taxonomy of the *X. translucens* species. We then scrutinized the genomes for major virulence features of these strains to identify genes that might be important for pathogenicity and in defining their host range.

## Materials and Methods

### Bacterial Strains, Growth Conditions, and DNA Extraction

The relevant data for all *X. translucens* strains used in this study are listed in Table 1. The genome sequence of *X. translucens* pv. *translucens* strain DSM 18974 was retrieved from the National Center for Biotechnology Information (NCBI) GenBank database (accession number LT604072). Strains LMG 726, LMG 727, LMG 728, LMG 730, LMG 843, and UPB458 were grown at 28°C on YDC agar medium (2% dextrose, 1% yeast extract, 2% CaCO3, 1.5% agar) for 48 h. Bacteria were then dissolved in 10 ml washing buffer (50 mM TRIS-HCl pH 8.0, 50 mM Ethylenediaminetetraacetic acid (EDTA) pH 8.0, 150 mM NaCl). The genomic DNA was then extracted with the NucleoSpin ^®^ Microbial DNA kit (Macherey Nagel, Duren, Germany), according to the manufacturer’s recommendations. Strains CFBP 2055, CFBP 2539, CFBP 2541, and CFBP 8304 were grown at 28°C on PSA medium (0.5% peptone, 2% sucrose, 1.5% agar) for 24 h. Bacteria were then resuspended in 10 mM MgCl_2_ and diluted to an optical density at 600 nm of 1.0. Cells from 2 ml were harvested by centrifugation and washed once with 10 mM MgCl_2_, and genomic DNA was isolated using QIAGEN Genomic tip 100/G (QIAGEN, Hilden, Germany) according to the manufacturer’s instructions. The genomic DNA from strain ICMP 16317 was extracted following a standard phenol/chloroform method (Booher et al., 2015).

**TABLE 1:** *X. translucens* pathotype strains used in this study and characteristics of the obtained genome assemblies.

Pathovar	Strain	Country	Isolated from	Origin^a^	Coverage	Assembly length	Plasmid length	Accession
*arrhenatheri*	LMG 727	Switzerland	*Arrhenatherum elatius*	BCCM	103	4,843,101	NA	CP086333
*cerealis*	CFBP 2541	United States	*Bromus inermis*	CFBP	404	4,504,942	NA	CP074364
*graminis*	LMG 726	Switzerland	*Dactylis glomerata*	BCCM	81	4,678,781	NA	CP076254
*hordei*	UPB458^b^	India	*Hordeum vulgare*	BCCM	94	4,679,124	NA	CP076249
*phlei*	LMG 730	Norway	*Phleum pratense*	BCCM	87	4,569,024	NA	CP076251
*phleipratensis*	LMG 843	United States	*Phleum pratense*	BCCM	121	4,902,099	NA	CP086332
*pistaciae* (group A)	CFBP 8304	Australia	*Pistacia vera*	CFBP	301	4,599,174	NA	CP074365
*pistaciae* (group B)	ICMP 16317	Australia	*Pistacia vera*	ICMP	161	4,386,175	NA	CP083804
*poae*	LMG 728	Switzerland	*Poa trivialis*	BCCM	86	4,792,655	NA	CP076250
*secalis*	CFBP 2539	Canada	*Secale cereale*	CFBP	186	4,565,955	NA	CP074363
*translucens*	DSM 18947^c^	United States	*Hordeum vulgare*	DSMZ	223	4,715,357	NA	LT604072
*undulosa*	CFBP 2055	Canada	*Triticum turgidum* subsp. *durum*	CFBP	379	4,653,288	46,036	CP074361-CP074362

*^a^DSMZ, German Collection of Microorganisms and Cell Cultures; CFBP, Collection of Plant Pathogenic Bacteria; ICMP, International Collection of Microorganisms from Plants; BCCM, Belgian Coordinated Collections of Microorganisms; UPB, Collection of plant pathogenic bacteria, Earth and Life Institute, UCLouvain.*

*^b^UPB458 is the name of the strain in the UPB collection from where it was obtained and corresponds to LMG 737 in the BCCM collection.*

*^c^Jaenicke et al. (2016).*

### Sequencing, Genome Assembly, and Annotation

The library preparation and DNA sequencing of strains LMG 726, LMG 727, LMG 728, LMG 730, LMG 843, and UPB458 were done at the Functional Genomics Center Zurich. For these strains, as well as for ICMP 16317, libraries were prepared and multiplexed with the PacBio SMRTbell ^®^ Express Template Prep Kit 2.0 (PacBio, Menlo Park, CA, United States) according to the published protocol^1^. Multiplex library preparation in pools of eight strains, including strains CFBP 2055, CFBP 2539, CFBP 2541, and CFBP 8304, and simultaneous sequencing of eight strains on one SMRTCell was conducted at the GENTYANE genotyping platform (INRA Clermont-Ferrand, France). All strains were sequenced with the PacBio Sequel technology.

Genomic sequences were then *de novo* assembled with Flye 2.7 for strains CFBP 2055, CFBP 2539, CFBP 2541, and CFBP 8304; Flye 2.8.1 for LMG 726, LMG 728, LMG 730, and UPB458; Flye 2.9 for LMG 727, and LMG 843; and HGAP 4 for ICMP 16317 (Chin et al., 2013; Kolmogorov et al., 2019). Assemblies produced with Flye were done using the “--plasmids --iterations 2” parameters. Genomes were functionally annotated using the NCBI Prokaryotic Genome Annotation Pipeline (PGAP) or with Prokka 1.13 (Seemann, 2014).

### Phylogeny and Comparative Analysis

Average nucleotide identity (ANI) was calculated using Pyani 0.2.11 with default parameters (Pritchard et al., 2016), and a phylogeny dendrogram was constructed using Ward’s hierarchical clustering method in R version 4.1.0. The gene content of the strains used in this study was compared with Roary 3.7.0 using the Prokka annotation with default parameters (Page et al., 2015). To identify genomic rearrangements and conserved genomic regions within the three clades that we defined, we compared the genomic structure of the complete genome sequences used in this study with Mauve v20150226 (Darling, 2004).

The presence of T2SS, T3SS, and T4SS was determined by tBLASTn using the amino acid sequences of the main components of each cluster retrieved from UniProt as query (Supplementary Dataset 1) and the genomic sequences of each strain as subject, with a 0.01 *e*-value threshold. The presence of each secretion system was then validated by looking for the presence of the cluster in the PGAP annotation at the predicted locus. The presence of T6SS was determined using SecReT6 3.0 with default settings (Li et al., 2015). The T3SS gene cluster was analyzed further by comparing the sequences of the PGAP-annotated genes using Clinker 0.0.21 with default parameters (Gilchrist and Chooi, 2021). In strains CFBP 8304 and LMG 726, the *xopF* gene found in the cluster was incorrectly annotated with PGAP, but was correctly annotated with Prokka, and was thus manually corrected in the PGAP annotation for the cluster comparison. The same was true for the *xopM* gene in strains UPB458, CFBP 8304, and LMG 728.

The presence of type III effectors was determined by BLASTp using the amino acid sequences of the effectors retrieved from http://xanthomonas.org/ as query (Supplementary Dataset 2) and the amino acid sequences of the genes annotated with Prokka as subject, with a 0.01 *e*-value threshold. Only hits that had > 30% identity over > 70% query sequence length were retained. To validate the presence of each effector, the amino acid sequences of the selected genes were then extracted and used as a query for a BLASTp against the type III effectors’ sequences as subject. Hits that had > 30% identity over > 70% query sequence length were then considered to be putative type III effectors, as discussed in this article. The presence of TALEs, their RVD sequence, and their classification were determined using AnnoTALE 1.5 (Grau et al., 2016).

## Results

### Genome Assembly

For all the sequenced strains, sequence coverages between 81- and 404-fold were obtained. This allowed to assemble complete genome sequences, consisting of one single circular chromosome for all strains (Table 1). Genome sizes ranged from 4,386,175 bp for ICMP 1317 to 4,902,099 for LMG 843. In strain CFBP 2055, an additional circular contig of 46,036 bp was assembled. A comparison by BLAST to the NCBI non-redundant nucleotide sequences database showed homology to plasmid sequences. This contig was thus considered to represent a plasmid of pv. *undulosa* strain CFBP 2055.

### Phylogeny and Comparative Analysis

**FIGURE 1:**
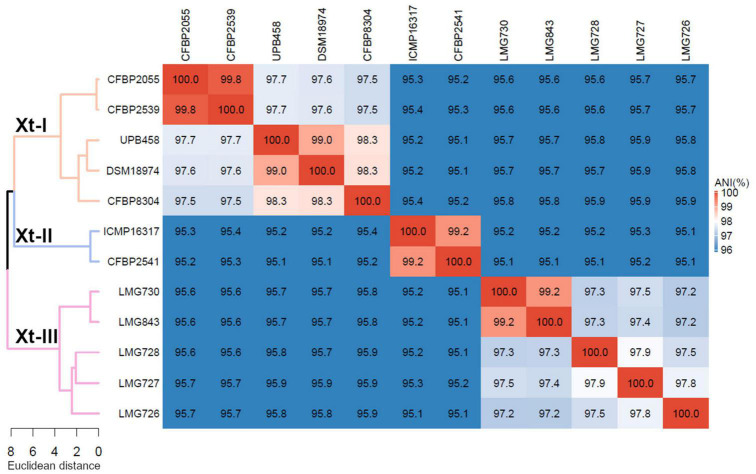
Average nucleotide identity (ANI) of *X. translucens* pathotype strains and ANI-based phylogeny constructed with Ward’s hierarchical clustering method. Distance in the dendrogram represents ANI dissimilarity between nodes. Orange: clade Xt-I, blue: clade Xt-II, pink: clade Xt-III. ANI is depicted as a gradient from blue (< 96%) to white (98%) to red (100%).

Phylogeny based on ANI revealed that though all strains shared > 95% ANI, three distinct groups could be observed, with ANI values above 97% (Figure 1). The first group consisted of strains CFBP 2055, CFBP 2539, UPB458, DSM 18794, and CFBP 8304; the second consisted of strains ICMP 16317 and CFBP 2541, and the third consisted of strains LMG 730, LMG 843, LMG 728, LMG 727, and LMG 726. These three groups will thereafter be referred to as clades Xt-I, Xt-II, and Xt-III, respectively. These three clades could also be observed when including all the publicly available complete genome sequences of *X. translucens* strains, with ANI values above 97% (Supplementary Figure 1). Interestingly, the two strains regarded as pv. *pistaciae* were genetically distinct and were found in two separate clades.

**FIGURE 2:**
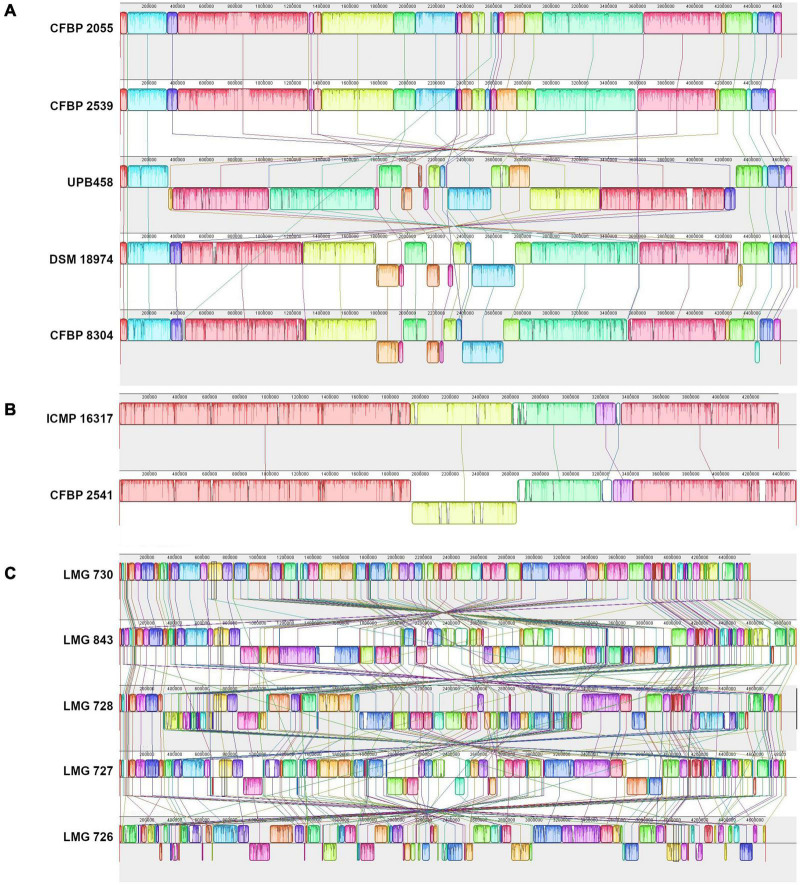
Pairwise comparisons of the genomic structure within the three *X. translucens* clades with progressive Mauve (Darling, 2004). **(A)** Clade Xt-I, **(B)** clade Xt-II, **(C)** clade Xt-III. Colors represent conserved genomic regions (locally collinear blocks, LCBs), i.e., regions with no rearrangement across all the compared genome sequences. Lines between strains link LCBs that are orthologous between two genome sequences. LCBs found on the bottom part represent regions that are in reverse orientation compared to the reference. In each comparison, the sequence on top is used as reference.

A comparison of the genomic structure within each clade showed 34–48 locally collinear blocks (LCB) in clade Xt-I (Figure 2A), 11 LCB in clade Xt-II (Figure 2B), and 175–312 LCB in clade Xt-III (Figure 2C). These LCB correspond to genomic regions that are conserved between the compared strains, showing no rearrangement. Thus, the low number of LCB in clade Xt-II showed that there are very few genomic rearrangements between the two strains of the clade, while there are more rearrangements in clade Xt-I and the most in clade Xt-III.

**FIGURE 3:**
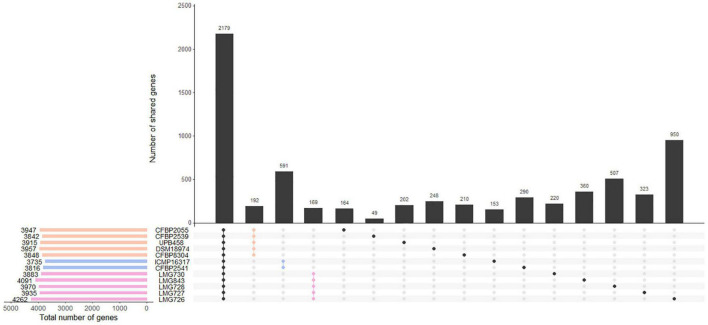
Gene content of the 12 *X. translucens* pathotype strains used in this study. Vertical bars show the number of genes exclusive to the strains marked in the matrix below. The first group represents the core genome of the species. The second, third, and fourth groups represent genes that are exclusive to the three clades, as indicated by their respective color. Other groups represent genes that are exclusive to one strain. Horizontal bars show the total number of genes in each strain. Orange: clade Xt-I, blue: clade Xt-II, pink: clade Xt-III.

The number of genes found in each strain with the Prokka annotation ranged from 3,735 genes in ICMP 16317 to 4,262 genes in LMG 726. The pangenome of the 12 *X. translucens* strains used in this study consisted of 9,772 genes, while the core genome consisted of 2,181 genes (Figure 3). A total of 190 genes were exclusive to clade Xt-I, 588 genes to clade Xt-II, and 168 genes to clade Xt-III. Moreover, a total of 3,681 genes were exclusive to one strain only, ranging from 48 genes in strain CFBP 2539 to 966 genes in LMG 726.

**FIGURE 4:**
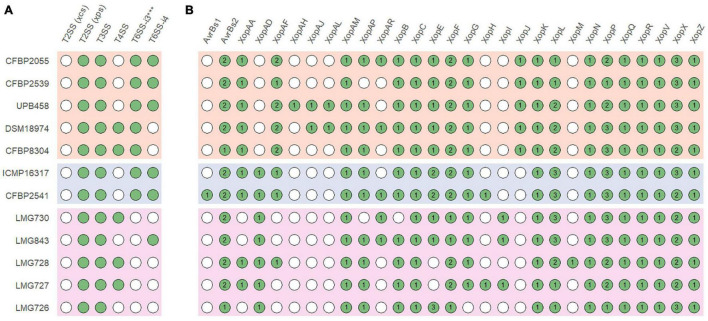
**(A)** Presence of the *xps* and *xcs* type II secretion systems (T2SS), type III secretion system (T3SS), type IV secretion system (T4SS), subtype 3*** type VI secretion system (T6SS-i3***), and subtype 4 type VI secretion system (T6SS-i4) gene clusters in *X. translucens* pathotype strains. **(B)** Presence of putative type III effectors. Green circles indicate presence, and white circles indicate absence. Numbers denote the number of putative effectors of each class. Orange: clade Xt-I, blue: clade Xt-II, pink: clade Xt-III.

In all strains, an *xps* T2SS and a T3SS were identified, while no *xcs* T2SS was identified in any strain (Figure 4A). A T6SS-i3^∗∗∗^ was identified in all strains of clades Xt-I and Xt-II, while a T6SS-i4 was identified in strains CFBP 2055, CFBP 2539, UPB458, ICMP 16317, CFBP 2541, and LMG 843. A T4SS was identified in strains DSM 18974, CFBP 8304, LMG 730, LMG 728, and LMG 727 which all lacked a T6SS-i4. The strain LMG 726 was the only one that did not possess a T4SS or a T6SS.

**FIGURE 5:**
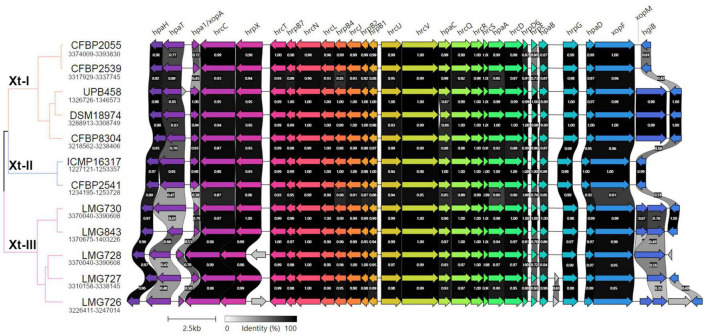
Pairwise comparisons of the genetic content of the type III secretion system cluster in the *X. translucens* pathotype strains obtained with Clinker (Gilchrist and Chooi, 2021). The phylogenetic tree is based on ANI as shown in Figure 1, orange: Clade Xt-I, blue: clade Xt-II, pink: clade Xt-III. Colors of the arrows represent groups of similar genes. Clusters for strains ICMP 16317, CFBP 2541, and LMG 727 are found in opposite direction and were reversed in the figure.

**FIGURE 6:**
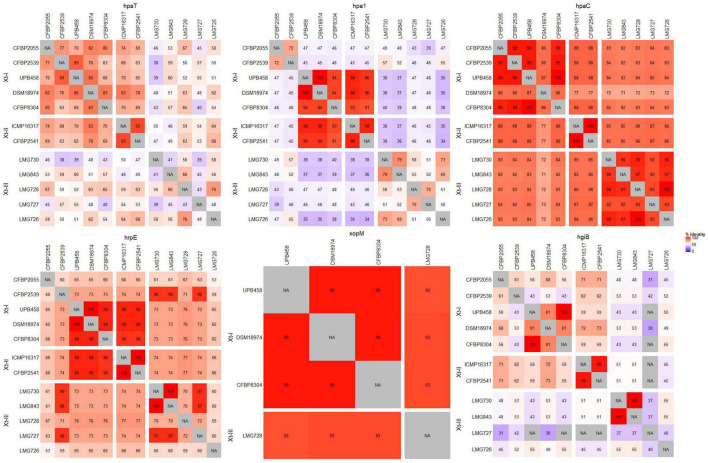
Pairwise comparisons of the nucleotide sequences of the most variable components of the type III secretion system across all *X. translucens* pathotype strains. For *xopM*, only UPB458, DSM 18974, CFBP 8304, and LMG 728 were considered, as they were the only strains where a complete gene was identified.

A direct comparison of the *hrp* cluster showed that all strains share the same genetic organization of the cluster (Figure 5). Moreover, most of the main components of the T3SS, from *hrcC* to *hrpD*, as well as *hpaH* and *xopF* were very conserved, with > 80% identity across the species. However, the *hrpE* structural component was the most variable, with as little as 60% identity within clades Xt-I and Xt-III (Figure 6). In strains LMG 728 and LMG 726, an additional gene was found between *hrpX* and *hrcT*, in opposite directions in each strain, but showing 100% identity. This gene showed a high identity with insertion sequence (IS) 5 family transposases found in other *Xanthomonas* species by BLASTx against the NCBI non-redundant protein sequence database. Moreover, in strains LMG 727 and LMG 726, one additional gene was predicted between *hpaB* and *hrpG*, with 61% identity between the two strains. However, no similarity to a known gene was found by BLASTx. Additionally, *hpaC* was found to be truncated in DSM 18974 due to an early stop codon, but still showed between 71 and 86% identity with the other strains (Figure 6).

The T3SS-associated components upstream and downstream of the core *hrp* cluster (*hpaT*, *hpa1*, *xopM*, and *hgiB*) showed more variability. The putative translocon *hpaT* was very conserved between the two clade Xt-II strains and showed between 63 and 89% identity in clade Xt-I (Figure 6). It was much more variable in clade Xt-III, where it showed between 39 and 67% identity. The second putative translocon component, *hpa1*, was very conserved among the two clade Xt-II strains (98% identity) and among the UPB458, DSM 18974, and CFBP 8304 strains in clade Xt-I. It was, however, more dissimilar between these three strains and the other two of the clade, with 45–47% identity. Within the Xt-III clade, there was a high variability, with 50–79% identity between strains. Moreover, one additional gene was predicted between *hpaT* and *hpa1* in UPB458, but no similarity to any known gene was found by BLASTx.

In clade Xt-I, the XopM effector was only present in UPB458, DSM 18974, and CFBP 8304 and was 99% identical between the three strains. The effector was also found in LMG 728, where it was 92–93% identical to the ones found in clade Xt-I. In the other four strains of clade Xt-III, the gene was disrupted by early stop codons. The *hgiB* gene was also very variable, with as low as 43% identity within clade Xt-I, and 37% identity within clade Xt-III, but with 99% identity between the two clade Xt-II strains. In LMG 728, it was found to be truncated. Furthermore, an additional gene was found between *xopM* and *hgiB* in LMG 726, which showed 100% identity to an IS4 family transposase by BLASTx.

Together with XopF and XopM, a total of 29 putative non-TALE type III effector classes were found to be present in the 12 *X. translucens* strains used in this study, ranging from 21 effectors in LMG 726 to 31 in CFBP 2541 and DSM 18974 (Figure 4B). Among the predicted type III effectors, AvrBs2, as well as effectors of classes XopC, XopF, XopK, XopL, XopN, XopP, XopQ, XopR, XopV, XopX, XopZ, and XopAM were found in all strains. The effectors of classes XopB, XopG, XopE, XopAA, and XopAF were conserved in all strains of clades Xt-I and Xt-II but present in only some strains of clade Xt-III. XopJ was found only in clade Xt-I, while XopAD was found only in clades Xt-II and Xt-III. Interestingly, the *xopM* gene located downstream of the *hrp* cluster was not found in this analysis, the XopM effector identified in LMG 728 being a different one. However, this XopM was found to be present in some strains by visual inspection of the gene clusters (Figure 5).

A total of 21 TALE classes were identified in the 12 strains used in this study, with up to 8 in DSM 18974 (Table 2). Clade Xt-I was the clade with the most TALEs. However, the TALE repertoires of the clade were very diverse, with only TalDA being present in all strains of the clade. Interestingly, TalDA was also found in ICMP 16317. Clade Xt-II and Xt-III strains had a much smaller set of TALEs, ranging from zero to three, with most of them being exclusive to one strain, except for TalIT, which was found in both LMG 843 and LMG 727. Additionally, a potential pseudo-TALE was identified in LMG 727, with only two repeats. However, the second repeat is only 19 amino acids long and many stop codons were found in the N-terminus. LMG 726 was the only strain in which no TALEs were identified.

**TABLE 2:** Classes and repeat variable diresidue (RVD) sequences of transcription activator-like effectors (TALEs) found in the *X. translucens* pathotype strains.

TALE class^a^	Strain	RVD sequence	Chromosome position (location:strand)
TalCT	CFBP 2055	HN-HD-HD-HD-NI-NI-NI-HN-HD-HD-NN-NN-NI-NN-HD	1,324,426–1,327,651:1
	CFBP 2539	NN-HD-HD-HD-NI-NI-NI-HN-HD-HD-NN-NN-NI-NN-HD	1,325,514–1,328,739:1
	DSM 18974	NN-HD-HD-HD-NI-NI-NI-NN-HD-HD-NN-NN-NI-NN-HD	671,336–674,561:1
	UPB458	NN-HD-HD-HD-NI-NI-NI-NN-HD-HD-NN-NN-NI-NN-HD	3,936,481–3,939,712:-1
TalCU	DSM 18974	NG-HD-HD-HN-NG-NI-HG-HG-HD-ND-NN-NN-NI-NH-QD	3,565,727–3,568,955:-1
TalCV	DSM 18974	NG-NN-HD-HD-NN-NI-HG-HD-ND-HG-NI-NN-HD	3,562,396–3,565,408:-1
	UPB458	NG-NN-HD-HD-NN-NI-HG-HD-ND-HG-NI-NN-HD	1,087,442–1,090,460:1
TalCW	DSM 18974	NN-NI-HN-HD-NI-NH-NG-HN-HD-HD-HD-NI-QD	660,311–663,329:1
TalCX	CFBP 8304	NN-HD-NG-NN-HN-KG-NI-HD-NI-NK-HD-HD-HD-HD-NI-HN-NH-HD-QD	674,110–677,491:1
	DSM 18974	NN-HD-NG-NI-HN-KG-NI-HD-NI-NH-NG-NN-HD-HD-NI-NN-NI-HD-QD	663,647–667,295:1
	UPB458	NN-QD-NG-NN-HN-KG-NI-HD-NI-NH-NG-HN-HD-HD-NI-NN-HD	3,939,840–3,943,281:-1
TalCY	DSM 18974	NI-NG-HN-NN-HD-NG-ND-NK-QD-NH-QD	668,858–671,207:1
TalCZ	CFBP 2055	NH-NN-HD-NN-HD-NH-HD-YK-NG-NH-Y*-HD-NN-NI-NG-QD	1,916,521–1,919,857:-1
	CFBP 2539	NH-NN-HD-NN-HD-NH-HD-YK-NG-NH-Y*-HD-NN-NI-NG-QD	1,917,023–1,920,359:-1
	DSM 18974	NH-NN-HD-NN-HD-NH-HD-YK-NG-NH-Y*-HD-NN-NI-NG-QD	1,995,271–1,998,607:-1
TalDA	CFBP 2055	HD-YD-NI-NG-NG-NN-YK-NG-HD-NG-NG-ND-NG-QD-NH-HD	593,184–596,364:1
	CFBP 2539	HD-YD-NI-NG-NG-NN-YK-NG-HD-NG-NG-ND-NG-QD-NH-HD	594,296–597,476:1
	CFBP 8304	HD-YD-NI-NG-NG-NN-YK-NG-HD-NG-NG-ND-NK-QD-NH-QD	653,260–656,587:1
	DSM 18974	NN-HD-NG-NG-NG-NN-YK-NG-HD-NG-NG-ND-NG-HD-NH-HD	627,243–630,573:1
	ICMP 16317	HD-YD-NI-NG-NG-NN-YK-NG-HD-NG-NG-ND-NG-QD-NH-HD	586,137–589,323:1
	UPB458	NN-HD-NG-NG-NG-NN-YK-NG-HD-NG-NG-ND-NG-HD-NH-HD	4,001,560–4,004,884:-1
TalDC	CFBP 2055	NN-NG-HD-HD-HD-KG-NN-Y*-NG-HD-HD-QD-HN	1,321,073–1,324,088:1
TalDD	CFBP 2055	NN-HD-NG-NN-HN-KG-NI-HD-NI-NN-HD-HN-HD-HD-NI-HN-HD-QD	612,552–616,092:1
	CFBP 2539	NN-HD-NG-NN-HN-KG-NI-HD-NI-NN-HD-HN-HD-HD-NI-HN-HD-QD	613,664–617,204:1
	CFBP 2539	NN-HD-NG-NN-HN-KG-NI-HD-NI-NN-HD-HD-NN-NN-NI-HN-HD	1,321,735–1,325,176:1
TalDE	CFBP 2539	NN-HD-NG-NN-HN-HN-NI-NI-NI-NH-NN-HD-NN-NH-HD-HD	1,717,212–1,720,551:1
	CFBP 2055	NN-HD-NG-NN-HN-HN-NI-NI-NI-NH-NN-HD-NN-NH-HD-HD	1,716,713–1,720,052:1
TalDF	CFBP 2055	HD-HN-HN-HD-NH-NH-HG-HD-ND-NN-Y*-NG-HD-NI-NH-NG-HD-HN	1,711,799–1,715,336:1
	CFBP 2539	HD-HN-HN-HD-NH-NH-HG-HD-KG-NN-Y*-NG-HD-NI-NH-NG-HD-HN	1,712,298–1,715,835:1
TalDO	CFBP 2541	NN-NN-KI-NN-HD-NG-HD-NG-NG-NK-HD-HD-NN-QD-NG-QD	2,549,947–2,553,289:1
TalDP	CFBP 2541	NS-KI-NI-HD-NK-GI-HD-NK-HD-NN-HD-NK	590,471–593,369:1
TalJU	LMG 843	NS-NG-HD-HD-NN	1,670,023–1,673,728:-1
TalJS	LMG 727	NN-NG-NN-NG-HD-NK-NG-NI-DD-NK-HD-NG-NN-NI-NG-NN-HD-HD-QD	3,139,389–3,143,094:1
	LMG 843	NN-NG-NN-NG-HD-NK-NG-NK-DD-NK-DD-NG-NN-NI-NG-NN-HD-HD-QD	2,289,805–2,292,265:1
TalIY	UPB 458	NI-NG-HN-NK-HD-NH-HN-HD-HD-HD-HD-QD	1,816,261–1,819,174:-1
TalJB	CFBP 8304	NH-NN-HE-NK-HD-NK-HD-YK-NG-NH-Y*-HE-NI-NI-NG-QD	1,986,200–1,989,539:-1
TalJT	LMG 730	NN-NN-HK-HK-HD-HN-HD-NN	2,152,104–2,154,627:1
TalJV	LMG 728	NN-NI-HD-HD-HD-HD-KT-NG-NN-NN-KT-NI-NN-HD-NG-NG-NN-HD-NK	2,784,954–2,788,575:-1
TalJW	LMG 728	HK-HD-HN-NI-NG-HD-HN-NI-NG-HD-NG-NN-HN-HD-NG-NN-NI-HD-NG-NN-HD-HD-QD	2,798,245–2,802,361:-1
TalJX	LMG 728	NK-NG-NI-HD-NG-NN-NG-HD-NK-N*-NK-HD-NN-HD-NG-NN-NI-NG-HD-HD-NN-HD-HD-NN-HD-HD-QD	1,903,396–1,907,938:-1
Pseudo TALE	LMG 727	NI-NN	2,236,495–2,238,688:1

*^a^TALE classes based on AnnoTALE (Grau et al., 2016).*

*Thirteenth residue is missing.

## Discussion

In this study, we generated high-quality complete genome sequences of all pathotype strains of *X. translucens* and make them available as a community resource for in-depth comparative genome analyses within one of the most important pathogenic bacterial species. These are the first complete genome sequences for strains of the pathovars *arrhenatheri*, *graminis*, *hordei*, *phlei*, *phleipratensis*, *pistaciae*, *poae*, and *secalis*. These resources complement the complete genome of the pv. *translucens* pathotype strain DSM 18974 (Jaenicke et al., 2016), as well as the already available genome sequences of pv. *cerealis, translucens*, and *undulosa* strains.

Phylogeny based on ANI revealed that three genetically distinct groups can be identified, with members of each clade being less than 96% identical to members of the two other clades. This is in contrast with the usual distinction of only two groups, the “translucens” group and the “graminis” group but is in line with the previous studies suggesting that pv. *cerealis* could be genetically distinct from the other pathovars (Peng et al., 2016; Langlois et al., 2017; Shah et al., 2019). Based on this phylogeny, we propose to classify these groups as clade Xt-I, containing pathovars *hordei, translucens*, *undulosa*, and *secalis*; clade Xt-II, containing pv. *cerealis*; and clade Xt-III, containing pathovars *arrhenatheri*, *graminis*, *phlei*, *phleipratensis*, and *poae*.

Moreover, the two strains of pv. *pistaciae* were grouped in two different clades, with the group A strain found in clade Xt-I and the group B strain found in clade Xt-II, the two strains being only 95.4% identical. This confirmed the previous phylogeny based on *gyrB* sequences where the group A strain was more closely related to pathovars *translucens, secalis*, and *undulosa*, while the group B strain was closest to pv. *cerealis* (Giblot-Ducray et al., 2009). There is clear evidence that these strains are not directly related, although they share the same host.

These results raise some limitations of the classical pathovar classification used in *Xanthomonas*. Indeed, the pathotype strains of pathovars *secalis* and *undulosa* are 99.8% identical and share similar genomic organization and virulence features. As they also have a similar host range, this could lead to them being considered as the same taxonomic entity. On the other hand, the two groups of pv. *pistaciae* are very different genetically and considering them under the same pathovar could hinder the better understanding of their respective biology. Despite these limitations, the pathovar classification was surprisingly robust for the other pathovars. Nonetheless, this work suggests that this classification, currently based solely on pathogenicity tests, should be rethought to better reflect the genetic relationships between pathovars and their evolutionary history.

Within the three clades identified by the ANI-based phylogeny, we found 190, 588, and 168 genes that were specific to clade Xt-I, Xt-II, and Xt-III, respectively, as well as 48–966 genes that were strain specific. However, these numbers are probably biased by the small number of strains included in the comparison and including more strains would result in a smaller number of clade- and strain-specific genes. Nonetheless, these constitute a valuable list of genes that could shape the host range of each clade and/or strain.

All strains had an *xps* T2SS and no *xcs* T2SS, as well as a T3SS with a similar genetic organization, although a few genes of the *hrp* cluster were variable in sequence between strains. Interestingly, strains that possessed a T4SS did not possess a T6SS-i4 and vice versa. As these two secretion systems are both involved in antimicrobial activity, it is possible that their function is redundant in *X. translucens* and only one of them is required to play this role. However, the presence or absence of these two secretion systems does not necessarily reflect the phylogenetic relationships between the strains investigated in this study. Additionally, a T6SS-i3^∗∗∗^ was found in all strains of clades Xt-I and Xt-II. However, it was previously hypothesized that this subgroup of T6SS could be non-functional due to the lack of a PAAR and could be complemented by the presence of another subtype of T6SS (Bayer-Santos et al., 2019). Interestingly, no T4SS or T6SS was identified in strain LMG 726, which confirms the previous research that showed that many pv. *graminis* strains lack a T6SS, with only strains Xtg2, Xtg9, Xtg10, and NCPPB3709 found to harbor one (Hersemann et al., 2017).

We have identified a total of 13 type III non-TALE effector classes that could constitute a core set of effectors in *X. translucens*. Moreover, XopAD was not found in the strains of clade Xt-I in our analysis but was previously identified in strain DSM 18974 in different analyses, indicating that it could also be part of the *X. translucens* core set of type III effectors (Peng et al., 2016; Koebnik et al., 2021; Shah et al., 2021). Five additional effector classes constitute a core set of effectors in clades Xt-I and Xt-II, as well as XopJ, which was specific to clade Xt-I. Some effector classes were specific to one strain, such as AvrBs1 in pv. *cerealis* strain CFBP 2541, or XopAH in pv. *hordei* strain UPB458. Furthermore, XopAJ and XopAL class effectors were found in both pv. *translucens* and *hordei*. These effectors could have a role in the host specificity of each clade and/or pathovar. However, no type III effector has yet been functionally characterized in *X. translucens*, and the effectors identified in this study will need to be functionally validated to confirm their role in the pathogenicity of each pathovar.

High-quality genome assemblies allowed for the identification of the first TALE to be reported in grass-infecting *X. translucens* strains, as well as in pathovars *secalis*, *hordei*, and *pistaciae*. No TALE was found in pv. *graminis* in our analysis, confirming previous research where no TALE was identified in draft genomes of the pathovar (Wichmann et al., 2013). The TALEs identified in pv. *undulosa* correspond to those previously identified in other strains of this pathovar, except for Xt4699-Tal3, for which no similar TALE was found, as was the case in strain ICMP 11055 (Peng et al., 2016; Falahi Charkhabi et al., 2017). The TALE of class TalDC that we identified has the same RVD sequence as Xt4699-Tal8 and a similar sequence to ICMP 11055-Tal4b. Additionally, the TALE of class TalDD identified in CFBP 2055 and CFBP 2539 has a similar RVD sequence to ICMP 11055-Tal2. Furthermore, the TALEs identified in pv. *cerealis* strain CFBP 2541 correspond to the TALE previously identified in strain Nxtc01, with the TALE of class TalDP having a similar sequence to Nxtc01-Tal1 (Shah et al., 2019). As these four TALEs have previously been shown to have a role in virulence, the corresponding effectors we identified in this study could play a similar role in their respective strain (Falahi Charkhabi et al., 2017; Peng et al., 2019; Shah et al., 2019). However, these are the only TALEs that have been functionally characterized in *X. translucens* to date. Nonetheless, as most of the TALEs identified in this study are clade or strain specific, they could be essential components of host adaptation. Indeed, TALEs activate the transcription of plant genes by binding to their promoter region. As these sequences vary between plant species, TALEs must be adapted to target the specific sequence of that promoter in their host and thus reflect processes of co-adaptation between the bacteria and its host (Jacques et al., 2016). However, the lack of TALEs in pv. *graminis* indicates that other mechanisms play a role in *X. translucens* host speciation.

The pv. *pistaciae* group A pathotype strain shared a very similar genomic organization, T3SS cluster organization, and gene identity, as well as type III effector and TALE repertoires with the pathotype strains of pv. *translucens* and *hordei*. The same was true for the group B pv. *pistaciae* strain and the pv. *cerealis* pathotype strain, which were even more closely related. This could indicate that a very small set of genes is responsible for their ability to infect pistachio, as a previous study showed that pv. *translucens* strain DAR 35705 was unable to cause symptoms in pistachio and other Anacardiaceaea, while the pv. *pistaciae* strains of both groups were pathogenic in Anacardiaceaea and Poaceae (Marefat et al., 2006). However, no type III effector or TALE exclusive to either strain of pv. *pistaciae* has been identified in this study.

Although it had the biggest set of total genes, the pv. *graminis* strain LMG 726 was found to lack many virulence features such as the T4SS, the T6SS, as well as TALEs and had the smallest set of type III effectors. This is surprising, as this pathovar is known to be the most widespread and virulent grass-infecting *X. translucens* pathovar, with the largest host range in clade Xt-III. Indeed, while pv. *phlei* and pv. *phleipratensis*, pv. *arrhenatheri*, and pv. *poae* are restricted to the genera *Phleum*, *Arrhenatherum*, and *Poa*, respectively, pv. *graminis* can infect many grasses from genera such as *Agrostis*, *Alopecurus*, *Dactylis*, *Deschampsia*, *Festuca*, *Lolium*, *Phalaris*, *Phleum*, *Poa*, and *Trisetum* (Egli et al., 1975; Egli and Schmidt, 1982). Nonetheless, these results go along previous research that showed that strains of pv. *graminis* also lack a flagellum and have a distinct type IV pilus compared to other *X. translucens* pathovars (Hersemann et al., 2017). It was hypothesized that since pv. *graminis* is usually spread by mowing tools, the lack of a flagellum might not hinder its ability to spread in the plant, as flagellar motility is mostly necessary for the bacteria to reach points of entry in the plant. Similarly, it might encounter less competition with epiphytic microorganisms, and the presence of a T4SS and/or a T6SS might not be crucial for its survival. Additionally, as these features can act as elicitors of plant defense, their absence in pv. *graminis* could help to evade such defense mechanisms—an intriguing hypothesis that, however, has not yet been tested.

## Conclusion

In conclusion, our study substantially increased the number of complete genome sequences available for *X. translucens*, providing high-quality genomic resources for all the pathovars of the species. These sequences constitute a valuable basis for future studies investigating the phylogenetic relationships between *X. translucens* pathovars and other *Xanthomonas* species, as well as their key genetic features. The virulence features of *X. translucens* that we identified will help to better understand the biology of each clade and/or pathovar, and processes of adaptation to their respective hosts. The inclusion of the high-quality genome sequences of the additional strains of each pathovar in further comparative genomics studies will help in refining this list, laying the foundations for the development of new resistant cereal crop and forage grass cultivars.

## Data Availability Statement

The genome sequences generated in this study can be found on https://www.ncbi.nlm.nih.gov with accession numbers CP074361-CP074365, CP076249-CP076251, CP076254, CP083804, CP086332-CP086333, and LT604072

## Author Contributions

FG and RoK: design of the study with the help of RaK, CB, JJ, VR-R, and BS. FG, VR-R, RaK, and SC: genome sequencing and assemblies. FG: data analysis with assistance from VR-R. FG, RoK, RaK, JJ, VR-R, CB, SC, and BS: writing and reviewing. All authors contributed to the article and approved the submitted version.

## Conflict of Interest

The authors declare that the research was conducted in the absence of any commercial or financial relationships that could be construed as a potential conflict of interest.

## Publisher’s Note

All claims expressed in this article are solely those of the authors and do not necessarily represent those of their affiliated organizations, or those of the publisher, the editors and the reviewers. Any product that may be evaluated in this article, or claim that may be made by its manufacturer, is not guaranteed or endorsed by the publisher.

## Footnotes

1
https://www.pacb.com/wp-content/uploads/Procedure-Checklist-Preparing-Multiplexed-Microbial-Libraries-Using-SMRTbell-Express-Template-Prep-Kit-2.0.pdf

## Supplementary Material

Supplementary Figure 1 Average nucleotide identity (ANI) of all available complete genome sequences of *Xanthomonas translucens* strains and ANI-based phylogeny constructed with Ward’s hierarchical clustering method. Distance in the dendrogram represents ANI dissimilarity between nodes. Orange: clade Xt-I, blue: clade Xt-II, pink: clade Xt-III. ANI is depicted as a gradient from blue (<96 %) to white (98%) to red (100%).

Supplementary Dataset 1 Amino acid sequences of all secretion system components used to predict the presence of these secretion systems by tBLASTn.

Supplementary Dataset 2 Amino acid sequences of all type III effectors (T3Es) used to predict the presence of these effectors by BLASTp.
