# Supplementary figures and images for: Complete Genome Assemblies of All Xanthomonas translucens Pathotype Strains Reveal Three Genetically Distinct Clades
Source: Front Microbiol. 2022 Mar 2;12:817815. doi: 10.3389/fmicb.2021.817815 (PMC8924669; doi:10.3389/fmicb.2021.817815)

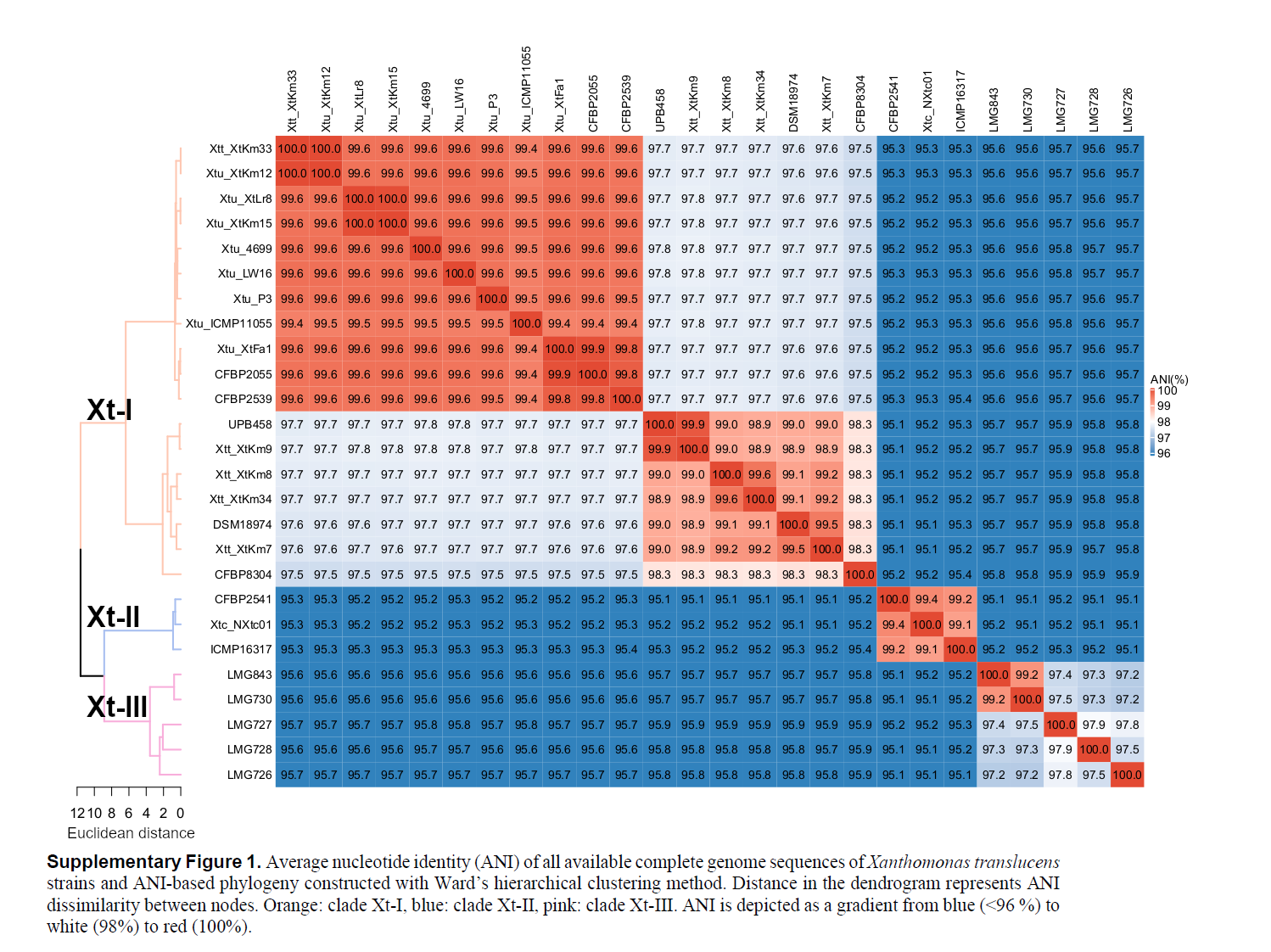

Supplement: Supplementary Figure 1 — Average nucleotide identity (ANI) of all available complete genome sequences of Xanthomonas translucens strains and ANI-based phylogeny constructed with Ward’s hierarchical clustering method. Distance in the dendrogram represents ANI dissimilarity between nodes. Orange: clade Xt-I, blue: clade Xt-II, pink: clade Xt-III. ANI is depicted as a gradient from blue (<96 %) to white (98%) to red (100%). [file Image_1.PNG]
